# Assessment of genotype‐trait interaction in maize (*Zea mays* L.) hybrids using GGT biplot analysis

**DOI:** 10.1002/fsn3.1826

**Published:** 2020-08-21

**Authors:** Seyed Habib Shojaei, Khodadad Mostafavi, Mahmoud Khosroshahli, Mohammad Reza Bihamta, Hossein Ramshini

**Affiliations:** ^1^ Department of Agronomy and Plant Breeding Science and Research Branch Islamic Azad University Tehran Iran; ^2^ Department of Agronomy and Plant Breeding Karaj Branch Islamic Azad university Karaj Iran; ^3^ College of Agriculture & Natural Resources (UCAN) University of Tehran Karaj Iran; ^4^ College of Agriculture & Natural Resources University of Tehran Pakdasht Iran

**Keywords:** corn, correlation coefficient, genotype–trait interaction, graphical method, PCA

## Abstract

In order to investigate the interaction of genotype × trait and relationships among agronomic traits on 12 maize hybrids, an experiment was conducted in a randomized complete block design (RCBD) with three replicates in four regions of Karaj, Birjand, Shiraz, and Arak. Results of analysis of variance indicated that most of the genotypes were significantly different in terms of agronomic traits. Mean comparison by Duncan's method showed that KSC705 genotype was more favorable than other genotypes in all studied regions. SC604 genotype in Birjand and Karaj regions and KSC707 genotype in Shiraz region have higher rank than other genotypes. Correlation analysis was used to investigate the relationships between traits. In most of the studied regions, traits of number of grains in row and number of rows per ear were positively and significantly correlated with grain width and grain weight with grain yield. Graphical analysis was used to further investigate. Genotypes–trait interaction graph explained 59.27%, 61.22%, 59.17%, and 61.95% of total variance in Karaj, Birjand, Shiraz, and Arak, respectively. Based on the multivariate graph, KSC705, KSC706, and SC647 genotypes were identified as superior genotypes in all studied regions and KSC400 genotype did not show much response to change in traits. Correlation between grain width and number of rows in ear, plant height and grain length, one thousand grain weight and grain thickness, and ear diameter with number of grains in row was positive and significant. The results of classification graph of genotypes also divided the cultivars in to three groups as follows: KSC703, KSC400, and KSC706 genotypes in the first group; DC370, SC604, and SC301 in the second group; and KSC260, KSC704, KSC707, and SC301 in the third group.

## Graphical abstract

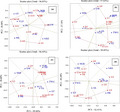

## 1 INTRODUCTION

The maize (*Zea mays* L.) is considered as one of the most important crop plants in the world, in such a way that it plays a considerable role in the provision of most of the world people's food (Panda, Behera, & Kashyap, 2004). According to the predictions, the demand for the maize in the developing countries would be twice the current demand until 2050 (Chaudhary, Kaila, & Rather, 2014). The corn breeding began since the human being has discovered the value of this plant in the provision of food, livestock, fiber, and fuel; and during the years, this plant has been changed from a wild plant to a crop one during the selection process by the farmers (Hallauer, Carena, & Miranda Filho, 2010). The main goal of plant breeding organizations is to identify the superior genotypes based on the multi‐environmental tests (MET) and the evaluation of different traits. The researchers evaluate several traits in different environments, but they usually encounter the problem while evaluating these traits. This problem occurs specially when there is negative interaction between the traits (De leon, Jannink, Edwards, & Kaeppler, 2016; Ghaghelestany, Jahanbakhshi, & Taghinezhad, 2020; Jahanbakhshi & Kheiralipour, 2019). In fact, the multi‐ environmental tests (MET) are carried out every year in the intended regions and the interpretation and effective use of analysis of these tests are so important in all the plant breeding stages such as trait‐based selection. The knowledge about the genotype (G) by environment (E) interaction can help the specification of superior genotypes based on the traits. Generally, many genotypes are tested in different times and places based on different traits and it often is hard to identify the superiority of genotypes yield in different environments. Different methods have been applied for the perception of the interaction, and these methods usually lead is resulted in a unit conclusion for a set of specific data. GGE biplot method permits the user to study and evaluate reciprocally the data (Dehghani, Dvorak, & Sabaghnia, 2012). The GGE biplot methodology is distinguished for being a versatile and flexible analysis allowing the selection of genotypes by means of graphical representations in an easy and efficient way (Yan, 2014). In GGE biplot method, Yan used two primary main elements which are resulted from the analysis of specific values on the data related to the yield of several environments. Anyway, this method has been introduced for the analysis of multi‐ spatial tests, but it can be used for any kind of data which has a reciprocal structure such as line tester, genotype by environment or genotype by trait (Yan, 2014). Yan and Rajcan (2002) used the genotype by trait (GT biplot) interaction method which is one of the GGE biplot methods for analyzing the genotype by trait data. This study revealed that GT biplot is an excellent tool for the identification of genotype by trait interactions. The GGE biplot method also has been used for evaluating the correlation of the traits by the genotype–trait biplot graphs (Akcura & Kokten, 2017). The identification of correlation between different traits and also the cause and effect relation between them help the breeders to select the most appropriate and logical relation between the constituents that is resulted in the further yield (Mardi, Talei, & Omidi, 2003). Kaplan, Kokten, and Akcura (2017) in studying of 25 silage maize, concluded that GGE biplot method with different perspective, could reliably by used in assessment of silage characteristics of maize genotypes grown in various environments. Adedeji, Ajayi, Osekita, and Ogunruku (2020) in studying of genotype × trait correlation on the cowpea cultivars, concluded that the majority of traits are positive and significant correlation with grain yield trait. In experiment done by Dolatabad, Choukan, Hervan, and Dehghani (2010) on the 14 maize hybrids in 9 research stations for studying genotype by trait (GT) biplot, clarified that correlation coefficient between grain yield components reveals a positive or negative relation between measured traits. Consequently, Gt biplot describes the interrelationships among traits and it was used to identifying hybrids that are good for some particular traits. Fan et al., (2007) in an experiment of 13 maize hybrids in 10 different regions used GGE biplot technique for studying traits correlation in various environments and concluded that yield stability should be useful in selecting superior hybrids in the absence of GGE biplot software. GGE biplot was also employed in variety evaluation of mung bean (Paramesh et al., 2016); green bean (Oliveira et al., 2018); maize (Setimela, Vivek, Banziger, Crossa, & Maideni, 2007); rice (Stanley, Samante, Wilson, Anna, & Medley, 2005); and black bean (Rocha et al., 2020).

In this study, the below aims are understudied:

Estimate the level of genetic variability among 12 cultivars of corn.
The study of genotype–trait interaction analysis which may be used for corn improvement program.
Choosing of the best hybrids according to the traits in the understudied regions.
The study of correlation between traits and the relations among them.
Grouping the genotypes based on the understudied traits in various regions.

## 2 MATERIAL AND METHODS

### 2.1 Experimental location and Maize hybrids

In this research, 12 maize hybrids were examined for investigating the genotype × trait interaction and the relationship between the crop traits base a randomized complete block design (RCBD) with three replications in four regions. Experiments were conducted in Karaj (50° 54'E, 35° 55'N), Birjand (59° 12'E, 32° 52'N), Shiraz (52° 36'E, 29° 32'N), and Arak (49° 46'E, 34° 06'N).

### 2.2 Experimental design

In this study, every experimental plot was designed with 4 rows, with 75 cm distance from each other. The seeds were planted with 10 cm distance from each other, and during the crop season, all the harvest operations such as the irrigation, weeding, and thinking were done consecutively and different crop traits were recorded. Also, irrigation system was similar for all experimental location. The names and codes of hybrids are represented in Table 1. Average of annual rainfall and codes and geographical parameters of under studied regions are represented in Table 2.

**Table 1:** Names and code of maize varieties studied in the experiment

Genotype No.	Genotype	Genotype No.	Genotype
G1	KSC703	G7	KSC707
G2	KSC260	G8	SC307
G3	KSC705	G9	SC647
G4	KSC400	G10	SC302
G5	KSC706	G11	SC604
G6	KSC704	G12	SC301

**Table 2:** Annual rainfall mean and codes and geographical parameters for the environments

Area	Longitude	Latitude	Elevation AMSL (m)	Average rainfall (mm)
Karaj	50° 54'E	35° 55'*N*	1,312	51.00
Birjand	59° 12'E	32° 52'*N*	1,491	59.13
Shiraz	52° 36'E	29° 32'*N*	1,484	52.32
Arak	49° 46'E	34° 06'*N*	1708	49.40

### 2.3 Data collection

For collecting the data, plant height (PH), ear length (EL), ear diameter (ED), number of grain in row (NGR), number of rows in ear (NRE), grain width (GW), grain length (GL), grain thickness (GT), one thousand grain weight (TWG), and grain yield (YLD) were evaluated.

### 2.4 Data analysis

**Figure 1:**
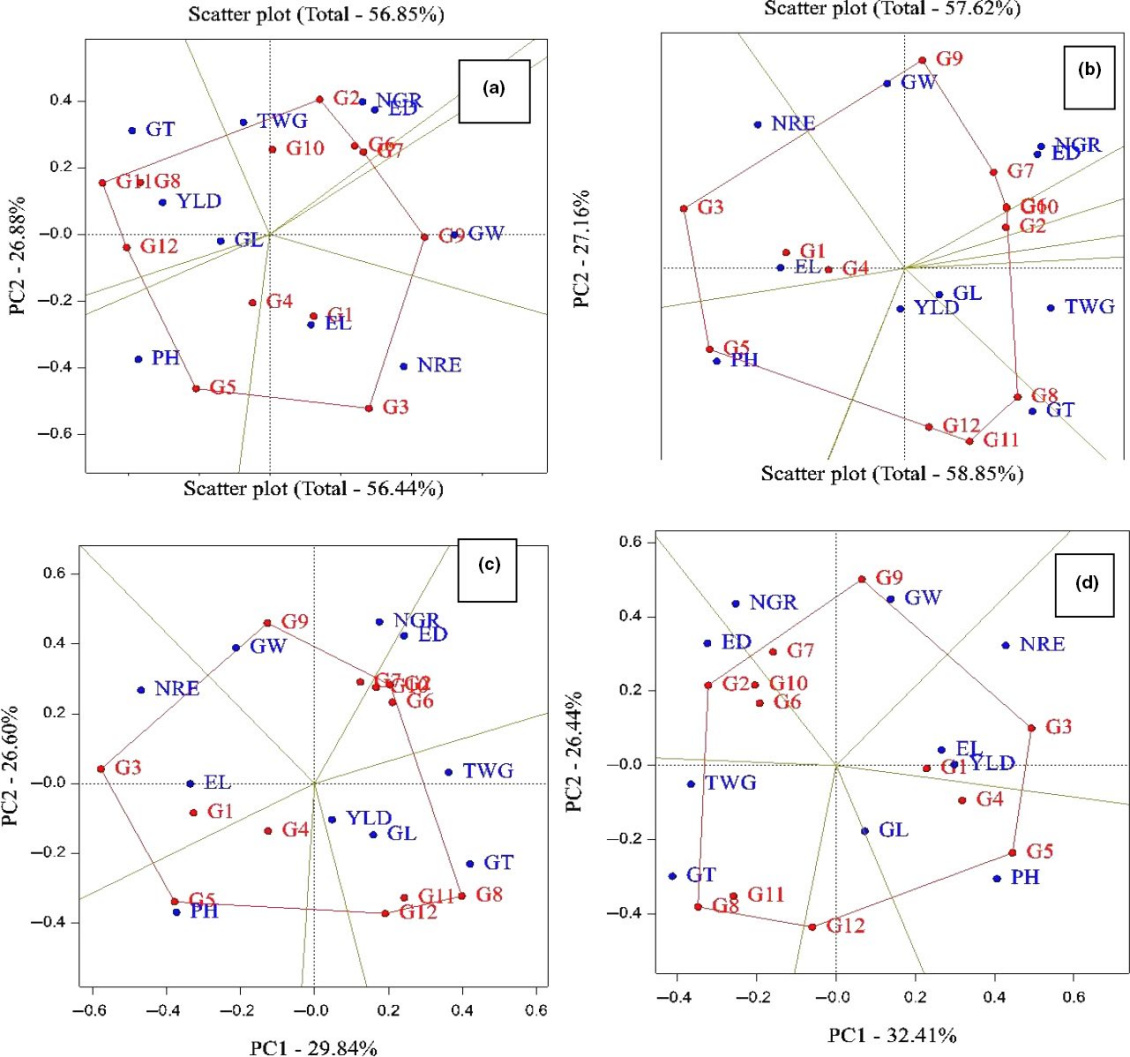
The polygon view for investigation of genotype–trait interaction. (a) Karaj station, (b) Birjand Station, (c) Shiraz Station, and (d) Arak Station. ED: ear diameter; EL: ear length; GL: grain length; GT: grain thickness; GW, grain weight; NGR, number of grains in row; NRE, number of rows in ear; PH, plant height; TWG, thousand grain weight; YLD, grain yield. G1: KSC703; G2: KSC260; G3: KSC705; G4: KSC400; G5: KSC706; G6: KSC704; G7: KSC707; G8: DC370; G9: SC647; G10: SC302; G11: SC604; G12: SC301

**Figure 2:**
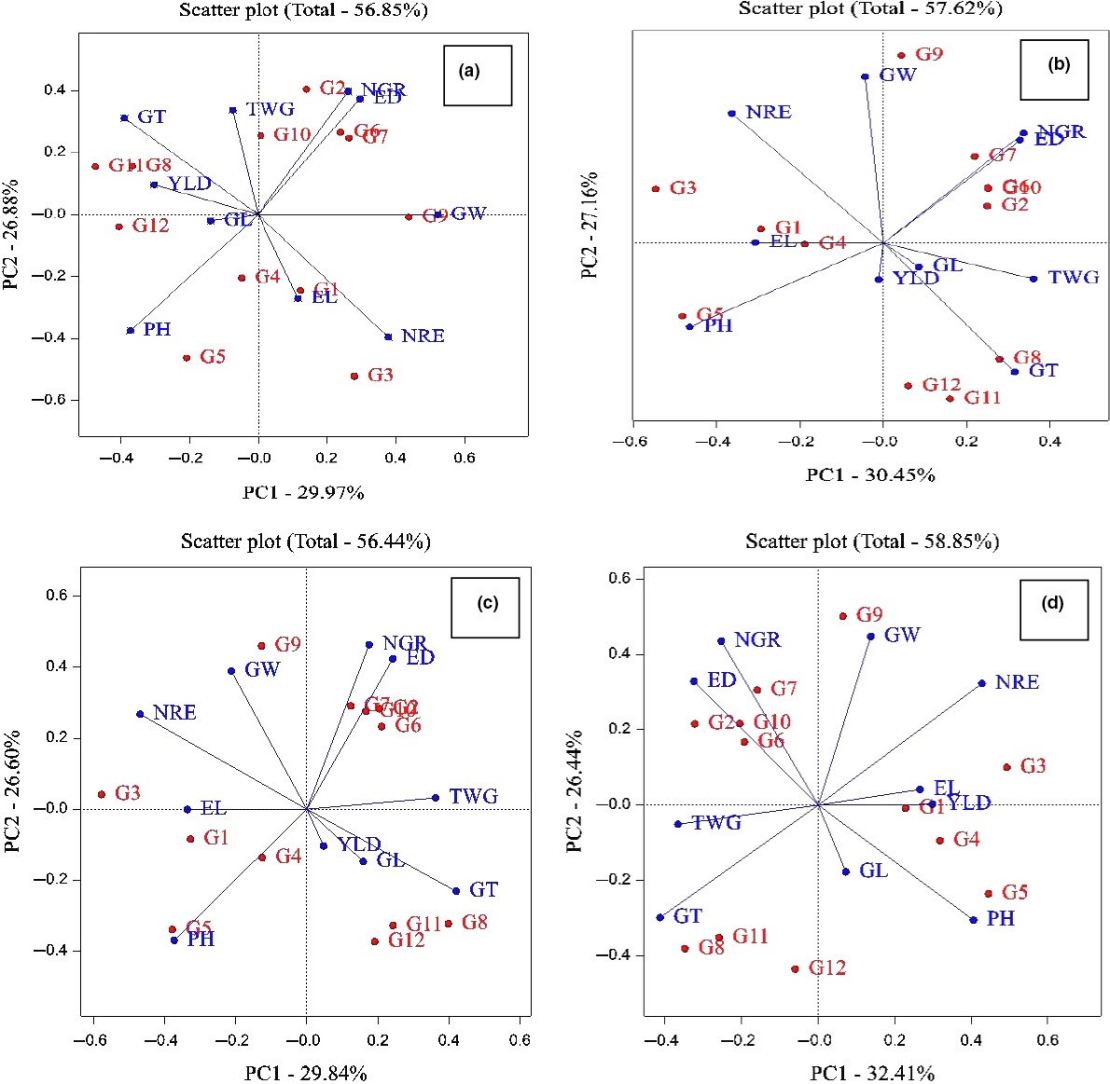
Correlation between different traits. (a) Karaj station, (b) Birjand Station, (c) Shiraz Station, and (d) Arak Station. ED, ear diameter; EL, ear length; GL, grain length; GT, grain thickness; GW, grain weight; NGR, number of grains in row; NRE, number of rows in ear; PH, plant height; TWG, thousand grain weight; YLD, grain yield; G1: KSC703; G2: KSC260; G3: KSC705; G4: KSC400; G5: KSC706; G6: KSC704; G7: KSC707; G8: DC370; G9: SC647; G10: SC302; G11: SC604; G12: SC301

**Figure 3:**
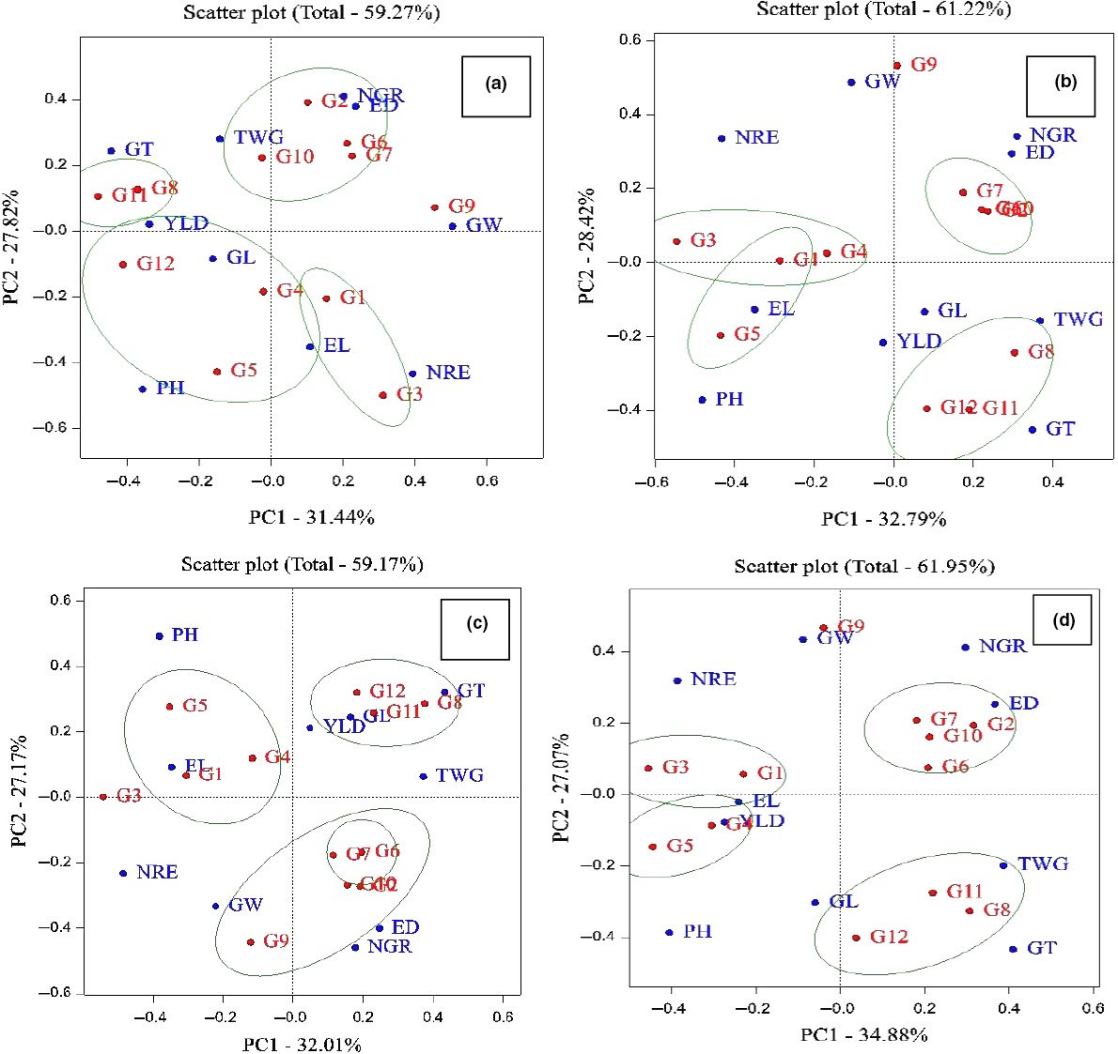
Classification between genotypes. (a) Karaj station, (b) Birjand Station, (c) Shiraz Station, and (d) Arak Station. ED, ear diameter, EL, ear length, GL, grain length, GT, grain thickness, GW, grain weight, NGR, number of grains in row; NRE, number of rows in ear; PH, plant height; TWG, thousand grain weight; YLD, grain yield. G1: KSC703; G2: KSC260; G3: KSC705; G4: KSC400; G5: KSC706; G6: KSC704; G7: KSC707; G8: DC370; G9: SC647; G10: SC302; G11: SC604; G12: SC301

Analysis of variance and other genetic parameters help to formulate a suitable breeding parameter and being prerequisites for any effective method of crop improvement (Osekita & Ajayi, 2013). Analysis of variance and mean comparison base Duncan multiple range test were used for investigation of traits in four environments. Relationships between different traits were examined using Pearson correlation coefficients. These analyzes were performed using SAS v.9.1 software for each environment. The genotype–trait interaction (GT biplot) investigated using principle component analysis. In this research, three biplot graphs were created with data matrix of the environment and genotypes by using GenStat software v12. These graphs are used for interpretation of relationship between genotypes and traits (Figure 1), correlation between of traits (Figure 2), and classification between genotypes and ranking of genotypes (Figure 3).

For studying the genotype × trait interaction, Yan and Rajcan (2002) method was used as below (Equation 1):

(1) $$\frac{\alpha_{\mathrm{ij}}‐\beta_{j}}{\sigma_{j}}=\sum_{n=1}^{2}\lambda_{n}\xi_{\mathrm{in}}\eta_{\mathrm{jn}}+\varepsilon_{\mathrm{ij}}=\sum_{n=1}^{2}\xi_{\mathrm{in}}^{*}\eta_{\mathrm{jn}}^{*}+\varepsilon_{\mathrm{ij}}$$

Where α_ij_: average amount of genotype *i* for every trait *j*, *β_j_*: average amount of all the genotypes for the traits, *σ_j_*: standard deviation of the trait *j* in the average genotypes ε*_ij_*: amount of genotype *i* remained in the trait *j*, *λ*_n_: certain amount for the main element (PC_n_), ξ_i_: amount of PC_n_ for the genotype *i*, and η_jn_: amount of PC_n_ for the genotype *j*.

## 3 RESULT AND DISCUSSION

Analysis of variance indicated that the genotype effect was significant for ear length, ear diameter, number of grains per row, grain width, and grain yield in all locations. Genotype effect was significant for thousand grain weight in Shiraz and Karaj. Genotype effect was significant differences for grain thickness in Arak and Birjand. The most and least percentage of coefficient of variation was related to the grain thickness and ear length, respectively (Table 3).

**Table 3:** Analysis of variance for traits of 12 maize hybrids in 4 environments

State	MS											
	S.O.V.	DF	BH	EL	ED	NGR	NRE	GW	GL	GT	TWG	YLD
KARAJ (E1)	Block	2	110.1ns	0.06^ns^	9.59^ns^	4.48^ns^	31.6^ns^	0.57^ns^	1.31^ns^	1.27^ns^	277.6^ns^	0.21^ns^
	Genotype	11	519.2^ns^	10.8^**^	62.6^**^	9.48*	73.79^ns^	2.91^**^	5.95^ns^	2.21^ns^	3,961.9*	0.77*
	CV%	–	9.91	7.80	9.28	11.46	19.30	15.81	228	30.16	18.9	18.7
BIRJAND (E2)	Block	2	247.6^ns^	0.04^ns^	8.1^ns^	14.9^ns^	193.1^ns^	0.64^ns^	3.82^ns^	2.82^ns^	2,899.7^ns^	9.59^ns^
	Genotype	11	516.4^ns^	7.06^**^	53.5^**^	6.83^**^	56.9^ns^	2.57^**^	6.36^ns^	2.21*	3,758.2^ns^	10.7*
	CV%	–	13.43	7.80	9.30	11.45	22.38	15.81	24.29	30.3	19.2	20.2
SHIRAZ (E3)	Block	2	193.4^ns^	15.1^ns^	352.1^ns^	7.15^ns^	106.6^ns^	0.40^ns^	2.92^ns^	3.62^ns^	477.2^ns^	4.81^ns^
	Genotype	11	659.1^ns^	6.1^**^	53.9^**^	6.77^**^	61.2^ns^	2.91^**^	7.14*	23.32^ns^	4,002.8*	68.3*
	CV%	–	12.75	8.09	11.71	11.59	22.08	17.14	22.18	30.63	20.65	26.20
ARAK (E4)	Block	2	59.4^ns^	13.3^ns^	513.6^ns^	1.19^ns^	139.3^ns^	1.84^ns^	1.87^ns^	2.02^ns^	1,329.3^ns^	0.21^ns^
	Genotype	11	649.4*	5.91^**^	61.6^**^	7.01*	65.9^ns^	1.74^**^	6.3^ns^	2.32*	3,858.6^ns^	0.77*
	CV%	–	13.07	8.16	11.77	11.67	21.23	19.11	23.3	38.33	20.91	18.87

Note

PH: plant height, EL: ear length, ED: ear diameter, NGR: number of grains in row, NRE: number of rows in ear, GW: grain weight, GL: grain length, GT: grain thickness, TWG: thousand grain weight, YLD: grain yield

* ,**, and ns: significant at 5%, 1%, and not significant.

Mean comparison base Duncan's method in all the studied regions indicated the genotype KSC705 has more utility in comparison to the other genotypes. In Birjand and Karaj, the genotype SC604, and in Shiraz, the genotype KSC707 had more superiority in comparison with the other genotypes. The genotype DC370 in Arak and Shiraz, the genotype SC647 in Shiraz and Birjand, and the genotype KSC706 in Karaj had the least rank in comparison with the other ones (Tables 4).

**Table 4:** Mean comparison for between traits in 12 hybrids of maize, cultivated in 4 environments

	Genotype	BH		EL		ED	NGR	NRE		GW		GL		GT		TWG		YLD	
KARAJ(E1)	G1	182ab		17.7b		38.4cde	15.1bc	39.1ab		6.3ab		9.4ab		2.9bc		258.4ab		7.1abc	
	G2	162.4b		17.1bc		42.3abcd	19.7a	35.6ab		5.5abc		6.8b		4.1ab		316.6ab		7.64ab	
	G3	197.8ab		21.4a		40.9bcd	15.1bc	48.6a		5.9a		8.4ab		2.7bc		290.5ab		6.65bc	
	G4	185.9ab		14.8cd		36.8de	14.5c	40.2ab		5.6ab		10.9ab		2.6bc		297.1ab		7.4ab	
	G5	198.7a		17.1bc		32.5e	14.6c	40.8ab		4.9bcd		8.6ab		3.7abc		225.8b		7.4ab	
	G6	167.3ab		16.8bcd		49.4a	16.7abc	37.7ab		5.8ab		10.2ab		4.3ab		297.8ab		6.35bcd	
	G7	171.9ab		16.8bc		42.9abcd	18.4ab	38.1ab		7.01a		10ab		3.6abc		336.6a		7.1abc	
	G8	190.8ab		14.4d		38.4cde	14.5c	30.5b		4.9bcd		10.2ab		4.6ab		337.4a		6.9bc	
	G9	176.6ab		14.4d		44.3abc	18.7ab	40.5ab		6.5ab		9.8ab		2.4c		232.6ab		6.42bcd	
	G10	176.2ab		15.6bcd		46.2ab	17abc	36.3ab		5.5abc		9.17ab		3.5abc		310.5ab		8a	
	G11	202.7a		16.1bcd		41.8abc	16.9abc	29.9b		3.6d		8.5ab		5.1a		276.6ab		7.4ab	
	G12	193.1ab		17.6b		37.5cde	15.9bc	35.2ab		3.9cd		12.4a		4.2ab		306ab		7.5ab	
BIRJAND(E2)	G1	166.3abc		14.2b		35.9cd	12.1b	31.6ab		5.9ab		10.5ab		3abc		245.7ab		6.3a	
	G2	149.2cd		13.8bc		39.2abc	15.7a	27.9ab		5.1abc		7.7b		4.2ab		303.2ab		4.8abc	
	G3	181.3a		17.2a		37.9bc	12.1b	38.6a		5.5ab		9.5ab		2.6bc		277.5ab		5.2ab	
	G4	170.9ab		11.9d		34.1cd	12.3b	31.5ab		5.3ab		11.7ab		2.6bc		283.4ab		4.8abc	
	G5	183a		13.8bc		30.1d	12.3b	32.9ab		4.6bcd		9.3ab		3.7ab		216.8b		5.6ab	
	G6	150bcd		13.5bcd		45.8a	14ab	30.5ab		5.5ab		11.4ab		4.3ab		286.7ab		5.1ab	
	G7	156.3bc		13.8bc		39.9abc	15.8a	29.6ab		6.5a		11.2ab		4.6ab		317.7ab		6.2ab	
	G8	156.6bc		11.6d		35.5cd	12.3b	23.9b		4.6bcd		11.2ab		4.6ab		332.9a		4.8abc	
	G9	144.8cd		11.6d		41abc	15.1a	35.3ab		6.1ab		10.7ab		2.4bc		232.7ab		5.2ab	
	G10	145.6cd		12.6bcd		14.3ab	14.3ab	31.3ab		5.2abc		10.6ab		3.5abc		309.4ab		6.1ab	
	G11	167.2ab		13bcd		38.7bc	13.5ab	23.7b		3.4d		9.2ab		5.1a		273.9ab		6.3a	
	G12	162.7abc		14.2b		34.8cd	12.7b	26.7ab		3.7cd		13.4a		4.3ab		305.8ab		5.9ab	
SHIRAZ(E3)	G1	192.6abc	14.02b		30.4cd		12.7d		33.3ab		5.8ab		10.1ab		3.1ab		265.6b		6.08abc	
	G2	173.1bc	13.4bc		36.7abc		16.6a		28.6ab		5abc		7.3b		4.3ab		328.1ab		4.7bc	
	G3	210.3ab	16.8a		35.2abcd		12.7d		39.5a		5.4ab		9.07b		2.7b		300.3ab		4.6bc	
	G4	198ab	11.69cd		31.7cd		12.9cd		32.3ab		5.2ab		11.3ab		2.7b		307.1ab		6.5abc	
	G5	211.8a	13.4bc		27.7d		13.03bcd		33.5ab		4.6bcd		9.02b		3.8ab		234.7b		6.5abc	
	G6	176.9bc	13.2bcd		42.3a		14.7abcd		31ab		5.5ab		11ab		4.5ab		310.1ab		5.7abc	
	G7	180.8abc	13.2bcd		37.4abc		16.1ab		30.7ab		6.6a		10.8ab		3.7ab		334.9ab		7.7a	
	G8	181.7abc	11.3d		33.1bcd		12.5d		24.4b		4.4bcd		11ab		4.8ab		348.8a		7.1ab	
	G9	168c	11.6cd		38.2abc		16.02abc		37.1ab		5.6ab		10.5ab		2.4b		241.2b		5.8abc	
	G10	168.6c	12.7bcd		40.2ab		14.5abcd		33.02ab		4.7bc		10.3ab		3.7ab		325.5ab		4c	
	G11	193.7abc	13.04bcd		35.6abc		13.7abcd		24.6b		3.1d		9.5ab		5.1a		284.7b		5.8abc	
	G12	188.3abc	13.4bc		32.1cd		12.9cd		27.8ab		3.4cd		13.6a		4.3ab		318.6ab		4.7bc	
ARAK(E4)	G1	191.2ab	13.7b		28.9ef		13.1bc		32.8ab		4.6ab		10.8ab		2.9ab		247.4ab		7.1ab	
	G2	172.02bc	13.2bcd		3.35abcde		16.8a		28.4ab		3.9abcd		7.9b		4ab		307.3ab		7.6ab	
	G3	210.1ab	16.6a		33.8abcde		12.9c		40.2a		4.3ab		9.7ab		2.5ab		281.3ab		6.6ab	
	G4	197.9ab	11.5cd		30.4edf		13.1bc		32.9ab		4.2abc		12.2ab		2.5ab		286.7ab		7.4ab	
	G5	215.5a	13.3bc		27.4f		13.1bc		34.1ab		3.7bcd		9.7ab		3.5ab		219.6b		7.4ab	
	G6	180.5abc	13bcd		41.8a		15abc		31.2ab		4.4ab		11.1ab		4.4ab		297.8ab		6.3b	
	G7	183.8ab	13bcd		37abcd		16.3a		30.7ab		5.2a		10.7ab		3.6ab		320.ab		7.1a	
	G8	184.7ab	11.1d		32.6cdef		12.6c		24.3b		3.5bcd		11ab		4.7ab		334.1a		6.9ab	
	G9	170.5bc	11.5cd		38.5abc		16.2ab		36.8ab		4.6ab		10.4ab		2.4b		231.4ab		6.4ab	
	G10	170.4bc	12.5bcd		40.6ab		14.7abc		33.1ab		3.9abcd		10.2ab		3.5ab		311.6ab		8a	
	G11	196.5ab	12.8bcd		36abcd		13.8abc		24.5b		2.6d		9ab		5a		272.1ab		7.4ab	
	G12	190.8ab	12.6bcd		31.9cdef		13bc		27.6ab		2.8cd		13.5a		4.1ab		304.4ab		7.5ab	

Note

ED, ear diameter; EL, ear length; GL, grain length; GT, grain thickness; GW, grain weight; NGR, number of grains in row; NRE, number of rows in ear; PH, plant height; TWG, thousand grain weight; YLD, grain yield.

G1: KSC703, G2: KSC260, G3: KSC705, G4: KSC400, G5: KSC706, G6: KSC704, G7: KSC707, G8: DC370, G9: SC647, G10: SC302, G11: SC604, G12: SC301

The correlation analysis was used for the recognition of the relations between the traits in this research. The results of coefficient of correlation between the traits in 12 maize hybrids studied in Karaj station revealed that the trait plant height has positive and significant correlation with the trait grain thickness. In addition, it had negative and significant correlation with ear diameter, number of grains in a row, and the grain width. The trait number of grains in a row also has positive and significant correlation with the trait grain width. The trait number of rows in ear also had positive and negative correlation with the traits grain width and grain thickness, respectively. On the other hand, the trait grain width had negative and significant correlation with the grain thickness. In addition, the traits grain thickness and weight of thousand grains respectively had positive and significant correlation with the traits the weight of thousand grains and yield (Table 5).

**Table 5:** Correlation coefficient between traits of 12 maize hybrids in Karaj region

	EL	ED	NGR	NRE	GW	GL	GT	TWG	YLD
PH	0.14^ns^	−0.44**	−0.33*	0.08^ns^	−0.34*	0.22^ns^	0.28^ns^	0.13^ns^	0.08^ns^
EL		−0.08^ns^	−0.1^ns^	0.2^ns^	−0.1^ns^	−0.01^ns^	0.06^ns^	−0.12^ns^	−0.08^ns^
ED			0.31*	−0.15^ns^	0.22^ns^	−0.14^ns^	−0.05^ns^	−0.11^ns^	−0.19^ns^
NGR				−0.07^ns^	0.33*	0.003^ns^	0.04^ns^	−0.11^ns^	−0.07^ns^
NRE					0.4**	−0.04^ns^	−0.39*	−0.004^ns^	−0.003^ns^
GW						−0.03^ns^	−0.31^ns^	0.01^ns^	−0.14^ns^
GL							0.03^ns^	0.1^ns^	0.1^ns^
GT								0.41**	0.26^ns^
TWG									0.46**

Note

ED, ear diameter; EL, ear length; GL, grain length; GT, grain thickness; GW, grain weight; NGR, number of grains in row; NRE, number of rows in ear; PH, plant height; TWG, thousand grain weight; YLD, grain yield.

* , **, and ns: significant at 5%, 1%, and not significant.

Analysis of correlation coefficient in Birjand station revealed the trait plant height had positive and significant correlation with the trait number of rows in ear. The trait ear length had negative and significant correlation with the grain width, weight of thousand grains, and grain yield. In addition, the ear diameter had negative and significant correlation with the trait grain width. Number of grains in a row, it had negative and significant correlation with the trait grain width and grain yield. The traits grain width and weight of thousand grains also had positive and significant correlation with the trait grain yield (Table 6).

**Table 6:** Correlation coefficient between traits of 12 maize hybrids in Birjand region

	EL	ED	NGR	NRE	GW	GL	GT	TWG	YLD
BH	0.29^ns^	−0.2^ns^	0.29^ns^	0.009*	−0.17^ns^	0.09^ns^	−0.2^ns^	0.03^ns^	−0.19^ns^
EL		0.12^ns^	0.39^ns^	0.26^ns^	−0.17*	0.24^ns^	−0.03^ns^	−0.21*	−0.32*
ED			−0.35^ns^	−0.27^ns^	−0.45*	0.27^ns^	−0.14^ns^	0.21^ns^	−0.22^ns^
NGR				0.23^ns^	−0.60**	0.08^ns^	−0.27^ns^	−0.03^ns^	−0.44*
NRE					−0.27^ns^	−0.09^ns^	0.01^ns^	−0.21^ns^	−0.25^ns^
GW						0.17^ns^	0.31^ns^	−0.03^ns^	0.41*
GL							0.11^ns^	0.32^ns^	−0.29^ns^
GT								0.14^ns^	0.12^ns^
TWG									0.05

Note

ED, ear diameter; EL, ear length; GL, grain length; GT, grain thickness; GW, grain weight; NGR, number of grains in row; NRE, number of rows in ear; PH, plant height; TWG, thousand grain weight; YLD, grain yield.

* ,**, and ns: significant at 5%, 1%, and not significant.

Based on the correlation analysis done between the traits in Shiraz station, the trait plant height had positive and significant correlation with the ear length. It had also negative and significant correlation with the ear diameter and number of grains in a row. The trait ear length also had negative and significant correlation with the trait ear diameter. Ear diameter had positive correlation with the number of grains in a row and negative correlation with grain yield. The trait number of grains in a row also had positive and significant correlation with the trait grain width. The trait number of rows in ear had positive and negative correlation with grain width and grain thickness, respectively. Additionally, the trait weight of thousand grains had positive and significant correlation with the grain yield (Table 7).

**Table 7:** Correlation coefficient between traits of 12 maize hybrids in Shiraz region

	EL	ED	NGR	NRE	GW	GL	GT	TWG	YLD
BH	0.28*	−0.44^**^	−0.33*	0.19^ns^	−0.04^ns^	−0.06^ns^	0.14^ns^	0.19^ns^	−0.002^ns^
EL		−0.32*	−0.22^ns^	−0.12^ns^	−0.09^ns^	−0.09^ns^	0.004^ns^	−0.17^ns^	−0.08^ns^
ED			0.32*	0.11^ns^	0.12^ns^	0.01^ns^	−0.17^ns^	0.02^ns^	−0.3^**^
NGR				0.12^ns^	0.44^**^	0.03^ns^	−0.02^ns^	0.05^ns^	−0.22^ns^
NRE					0.45^**^	−0.09^ns^	−0.35*	−0.08^ns^	−0.23^ns^
GW						−0.09^ns^	−0.26^ns^	0.02^ns^	0.05^ns^
GL							−0.02^ns^	0.08^ns^	−0.15^ns^
GT								0.36*	0.21^ns^
TWG									0.05*

Note

PH, plant height; EL, ear length; ED, ear diameter; NGR, number of grains in row; NRE, number of rows in ear; GW, grain weight; GL, grain length; GT, grain thickness; TWG, thousand grain weight; YLD, grain yield.

* , **, and ns: significant at 5%, 1%, and not significant.

The results of correlation coefficient between the traits in Arak region indicated the positive and significant correlation between plant height and grain width and grain yield. There was negative and significant correlation between the ear length and the trait ear diameter. The traits number of grains per row and number of rows per ear had positive and significant correlation with the grain width. Number of grain row in ear had negative correlation with grain thickness. The trait grain width had positive correlation with the grain length and negative correlation with grain thickness and grain yield. The trait weight of thousand grains also had positive and significant correlation with the trait grain yield (Table 8).

**Table 8:** Correlation coefficient between traits of 12 maize hybrids in Arak region

	EL	ED	NGR	NRE	GW	GL	GT	TWG	YLD
BH	0.16^ns^	−0.25^ns^	−0.27^ns^	0.23^ns^	0.02*	0.04^ns^	0.07^ns^	0.09^ns^	0.03^ns^
EL		−0.3*	−0.18^ns^	−0.08^ns^	0.22^ns^	0.04^ns^	−0.02^ns^	−0.23^ns^	0.06^ns^
ED			0.25^ns^	0.13^ns^	0.26^ns^	−0.27^ns^	−0.12^ns^	0.13^ns^	0.19^ns^
NGR				0.07^ns^	0.44^**^	0.005^ns^	0.04^ns^	0.05^ns^	−0.1^ns^
NRE					0.5^**^	−0.09^ns^	−0.36*	0.06^ns^	−0.14^ns^
GW						0.06*	−0.03*	0.05^ns^	−0.3*
GL							0.02^ns^	0.12^ns^	0.09^ns^
GT								0.37^ns^	0.27^ns^
TWG									0.39*

Note

ED, ear diameter; EL, ear length; GL, grain length; GT, grain thickness; GW, grain weight; NGR, number of grains in row; NRE, number of rows in ear; PH, plant height; TWG, thousand grain weight; YLD, grain yield.

* , **, and ns: significant at 5%, 1%, and not significant.

By studding the four understudied stations, it could be understand that trait thousand grain weight with grain yield, ear width with the number of grains in row, and number of grains in row with grain width had positive and significant correlation. Plant height with ear width and the number of grains in row, grain width with grain thickness, and number of rows in ear with grain thickness had negative and significant correlation. The result of this study was approximately similar to research of Dolatabad et al. (2010).

The graphical analysis was used for studying and interpreting the genotype by trait interaction. The polygon graph is used for determining the most appropriate genotype for every trait. This is resulted from the interconnection of the genotypes which have the most distance from the origin, in such a way that the other genotypes exist inside this polygon. A vertical line is drawing from the origin on every side of the polygon so that the shape be divided into several sector. In this graph, every genotype placed in a sector with a trait, would reveal higher utility and performance in that trait. Okoye, Okwuagwu, Uguru, Ataga, and Okolo (2007) and Dehghani, Omidi, and Sabaghnia (2008) had used this type of graph for studying rapeseed, and Dolatabad et al. (2010) had used this type of graph for studying maize. The results of graphical analysis done in Karaj station indicated that KSC705, KSC706, SC604, KSC260, and SC647 were recognized as the superior genotypes in this region. Also, the genotypes SC647, KSC705, and KSC706, respectively, had higher performance for grain width, number of rows in ear, and plant height and the DC370 genotype also showed higher performance in the traits grain thickness and grain yield. With regard to the nearness to the graph origin, the genotype KSC400 did not show a considerable reaction to different traits (Figure 1a).

In Birjand station, the genotypes SC604, DC370, KSC707, SC647, KSC705, and KSC706 have higher utility in comparison with the other genotypes. In addition, the genotypes KSC706 and KSC703 revealed further performance in the plant height and ear length, respectively. The genotypes DC370 and SC604 have further performance in the grain thickness in comparison with the other genotypes. Furthermore, in this station, with regard to this matter that the genotype KSC400 placed near to the origin, it revealed no considerable reaction to the change of traits. In addition, the traits grain length and grain yield showed no reaction to the nearness to the origin and change of the genotypes (Figure 1b).

In the study done in Shiraz station, the genotypes DC370, SC302, KSC260, SC647, KSC705, and KSC706 were selected as the superior genotypes. The genotypes DC370, SC647, and KSC706, respectively, revealed further performance in the grain thickness, grain width, and plant height in comparison with the other genotypes (Figure 1c).

In the polygon graphical analysis done in Arak region, the genotypes DC370, SC301, KSC706, KSC705, SC647, and KSC260 were selected as the superior genotypes. The genotypes DC370, SC647, and KSC706, respectively, had higher performance in the grain thickness, grain width and plant height in comparison to the other genotypes. The genotype KSC703 also had higher utility in the ear length and grain yield (Figure 1d).

It can be concluded that the genotypes KSC705, KSC706, and SC647 were recognized as the superior hybrids, since they had higher performance in comparison with the other genotypes in all the studied stations. As well, it was revealed that in all the studied stations, the genotypes DC370 and KSC706, respectively, had higher performance in the traits grain thickness and plant height in comparison with the other genotypes and the genotype KSC400 did not show considerable reaction to different traits.

The correlation graphical analysis was used for evaluating the correlation between the traits. In this biplot graph, the cosine of the angle between the traits vectors is indicative of the correlation intensity between the traits. If the angle between the two traits vectors be less than 90^˚^, equal to 90^˚^, and 180^˚^, the correlation between the vectors would be +1, 0, and −1, respectively. Kaplan et al. (2017) and Dolatabad et al. (2010) had used this type of graph for studying maize varieties, and Adedeji et al. (2020) had used this type of graph for studying cowpea. Accordingly, in Karaj region, the ear length, number of rows in ear, and grain width vectors had positive and significant correlation with each other since the angle between them was less than 90^˚^. So, they were categorized in one group. In addition, the traits "plant height and grain length," "grain yield, grain thickness, and one thousand grain weight," and "ear diameter and number of grains in row" were categorized in the second, third, and fourth groups, respectively. Accordingly, it can be concluded that the vectors of two traits grain thickness and number of rows in ear had negative and significant correlation with each other since they had 180^˚^ angle. In addition, the plant height, ear diameter, and number of grains in row had negative and significant correlation with each other (Figure 2a).

In Birjand region, with regard to the angle between the vectors, "grain thickness, grain length, and one thousand grain weight," "grain yield, plant height, and grain length," "number of rows in ear and grain width" and "ear diameter and number of grain in row," respectively, were categorized into the first, second, third, and fourth groups and had positive and significant correlation with each other. Since the traits "number of rows in ear with grain thickness" and "grain yield with grain width" has 180^˚^ angle between two vectors, they had negative and significant correlation with each other (Figure 2b).

In the study of Shiraz regions, "grain yield, grain length, and grain thickness," "plant height and grain length," "number of rows in ear and grain width," and "one thousand grain weight, ear diameter, and number of grain in row," respectively, were categorized into the first, second, third, and fourth groups and had positive and significant correlation with each other. In addition, with regard to this matter that the angle between two vectors in the traits number of rows in ear and grain thickness, grain width and grain yield, and one thousand grain weight and grain length was 180^˚^, they had negative and significant correlation with each other (Figure 2c).

Also, in Arak region, the "grain length, plant height, and grain yield," "ear length, number of rows in ear, and grain width," "ear diameter and number of grain in row" and "grain thickness and one thousand grain weight" were categorized into the first, second, third, and fourth groups and had positive and significant correlation with each other. In addition, the traits "one thousand grain weight and grain yield," "grain thickness and number of rows in ear," and "number of grains in row and grain length" had negative and significant correlation with each other (Figure 2d). It can be concluded that in all the studied regions, the traits "grain width and number of rows in ear," "plant height and grain length," and "ear diameter and number of grain in row" had positive and significant correlation with each other and the grain thickness and number of rows in ear had negative and significant correlation with each other.

For evaluating the classification between the genotypes in terms of the traits, the graph related to the classification between the genotypes was used (Figure 3).

Based on the Figure 3 which indicates the genotypes grouping from the data related to Karaj region, this graph explained 59.27% of the data total variance that 31.42% and 27.82% of the data variance were related to the first and second principal components, respectively. Based on the genotype ranking graph, the genotypes were categorized into four groups: The first group included the genotypes KSC704, KSC707, KSC260, and SC302; the second group included the genotypes DC370 and SC604; the third group included the genotypes SC301, KSC400 and KSC704, and the last group included the genotypes KSC703 and KSC705, genotype SC647 was not in the same group with other genotypes. (Figure 3a). In the study of data related to Birjand region, this graph explained 61.22% of the data total variance that 32.79% and 28.42% of that were related to the first and second principal components, respectively. The genotypes were categorized into four groups in this region: The first group included the genotypes DC370, SC604, and SC301; the second group included the genotypes KSC400, KSC703, and KSC705; and the third group included the genotypes KSC706 and KSC703. Genotype KSC 703 was present in the second and the third group. The last group included the genotypes KSC260, KSC704, and KSC707. Genotype SC647 was not in the same group with other genotypes (Figure 3b). In the study done on Shiraz station, 59.17% of data total variance was explained in this graph that 32.01% and 27.17% of that were respectively related to the first and second principal components. In this graph, the genotypes were categorized into four groups: The first group included the genotypes KSC400, KSC703, and KSC706; the second group included the genotypes DC370, SC604, and SC301; the third group included the genotypes SC647, KSC260, SC302, KSC704, and KSC707; and the last group included the genotypes KSC 704 and KSC707. Genotype KSC705 was not in the same group with other genotypes (Figure 3c). In the study done on Arak station, 61.95% of data total variance was explained by this graph that 34.88% and 27.07% of that were respectively related to the first and second principal components. The genotypes were categorized into four groups: The first group included the genotypes KSC360, KSC707, SC302, and KSC704; the second group included the genotypes KSC703 and KSC705; the third group included the genotypes KSC400 and KSC706; and the last group included the genotypes SC604, DC370, and SC301. Genotype SC647 was not in the same group with other genotypes (Figure 3d). With regard to the obtained data, it can be concluded that in all the studied regions, the genotypes "KSC703, KSC400, and KSC706," "DC370, SC604, and SC301," and "KSC260, KSC704, KSC707, and SC301," respectively, were categorized into the first, second, and third groups. KSC705 and SC647 were not in the same group with other genotypes. Dolatabad et al., (2010) had used this type of graph for studying maize.

## 4 CONCLUSION

GT biplot technique allowed essential and reliable assessment, examined traits in various environments. Based on this technique, it clarified how traits are changed in genotypes and different environments and describes the interrelationships among traits.
Result indicated that investigating different genotypes in various environments, KSC400, KSC706, and SC647, were identify superior hybrids. The traits grain thickness–one thousand grain weight, grain length–plant height, grain width–number of rows in ear, and number of grains in row–ear diameter had positive and significant correlation in the majority of the regions. Based on the classification between genotypes, the genotypes were categorized into three groups.
The highest grain yield in all locations belonged to *KSC707* cultivar at 6.9 t/ha followed by *SC604* with 6.7 t/ha.

## 5 INFORMED CONSENT

Written informed consent was obtained from all study participants.

## CONFLICT OF INTEREST

The authors have declared no conflict of interest.

## ETHICAL APPROVAL

This study does not involve any human or animal testing.
